# Structure and charge analysis of a cyclic alu­minium hy­dride: *cyclo*-1,5-bis-μ-di­methyl­amino-3,7-di-μ-hy­drido-2,4,6,8-tetra­kis­(di­methyl­alu­minium)

**DOI:** 10.1107/S2053229622011391

**Published:** 2023-01-01

**Authors:** Peter W. R. Corfield, Joshua Schrier

**Affiliations:** a Department of Chemistry, Fordham University, 441 East Fordham Road, Bronx, New York 10458, USA; Bristol-Myers Squibb, USA

**Keywords:** crystal structure, hy­dride bridge, organoalu­minium, DFT, charge determination

## Abstract

The title com­pound, [Al_4_(CH_3_)_8_(C_2_H_7_N)_2_H_2_], crystallizes as eight-membered rings with –(CH_3_)_2_Al–(CH_3_)_2_N–(CH_3_)_2_Al– moieties connected by single hy­dride bridges. In the X-ray structure, the ring has a chair conformation, with the hy­dride H atoms being close to the plane through the four Al atoms. An optimized structure was also calculated by all-electron density functional theory (DFT) methods, which agrees with the X-ray structure but gives a somewhat different geometry for the hy­dride bridge. Charges on the individual atoms were determined by valence shell occupancy refinements using *MoPro* and also by DFT calculations analyzed by several different methods. All methods agree in assigning a positive charge to the Al atoms, negative charges to the C, N, and hy­dride H atoms, and small positive charges to the methyl H atoms.

## Abstract 2

The title com­pound crystallizes as eight-membered rings with –(CH_3_)_2_Al–(CH_3_)_2_N–(CH_3_)_2_Al– moieties connected by single hy­dride bridges. The mol­ecular structures and partial atomic charges determined both by X-ray methods and by DFT calculations are compared.

## Introduction

In E. P. Schram’s early studies on the organometallic chemistry of alu­minium, his group analyzed the products of the reaction between di­methyl­amino­boranes and methyl alu­minium hy­drides (Hall & Schram, 1969 ▸; Schram & Hall, 1971 ▸; Schram *et al.*, 1969 ▸). In further work, the reaction of di­methyl­amino­bor­ane, [(CH_3_)_2_NBH_2_]_2_, with tri­methyl­alu­minium led to the isol­ation of a solid crystalline material. Analysis of single-crystal X-ray data collected in 1970–71 characterized the mol­e­cule that had been formed as *cyclo*-1,5-bis-μ-di­methyl­amino-3,7-di-μ-hy­drido-2,4,6,8-tetra­kis­(di­methyl­alu­minium), Al_4_(CH_3_)_8_[N(CH_3_)_2_]_2_H_2_, **1** (Scheme 1). The mol­ecule consists of an eight-membered ring containing singly-bridged hy­dride atoms, one of the first examples of such bridging at that time. Circumstances prevented completion of the refinement, although the mol­ecular structure without atomic parameters was described in a paper on the chemical reaction (Glore *et al.*, 1972 ▸). We now present details of this X-ray study based upon refinement of the 1971 data, together with an atomic charge–density analysis, and we compare the structure and charges with those found from a theoretical study.

## Experimental

### Synthesis and crystallization

Details of the vacuum line synthesis of the title com­pound, its purification by vacuum sublimation, and its chemical and spectroscopic analyses are given in Glore *et al.* (1972 ▸). The data crystal was mounted in a 0.5 mm thin-walled capillary tube in a dry-box and sealed under a nitro­gen atmosphere.

### Refinement details

Crystal data, data collection and structure refinement details are summarized in Table 1 ▸. Since the original data reduction listed structure factors, *F*, rather than the *F* ^2^ values used in today’s refinements, the 29 cases where the averaged values of the net intensity were less than zero had been recorded with *F* values of zero. On pre­paring *hkl* files with *F* ^2^ values, these 29 zero values were replaced with *F* ^2^ = σ(*F* ^2^) = 0.63*S*, where *S* was the average value of σ(*F* ^2^) for reflections with *F* ^2^ < 3σ(*F* ^2^) in the same θ range. 0.63*S* was chosen as the most probable value of the missing reflection. 
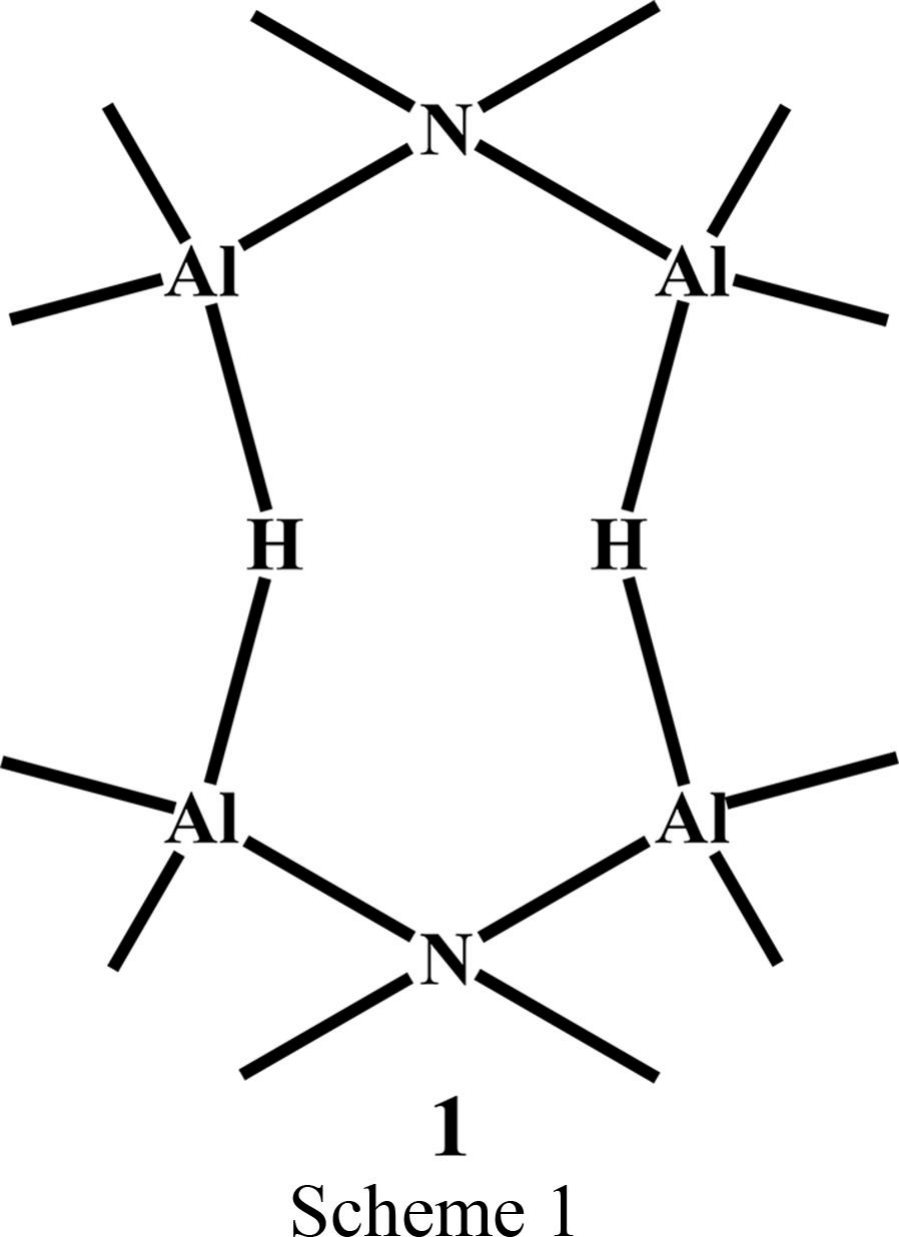


In refinements with *SHELXL2018*, the methyl H atoms were constrained to tetra­hedral geometry, with bond lengths of 0.96 Å and displacement parameters set to 1.5 times the average *U* value of the methyl C atom. A torsion angle was also refined for each methyl group. The bridging H atom was allowed to refine freely with an isothermal displacement parameter. Standard scattering factors were used and an extinction parameter was refined.

In the *MoPro* charge refinements, scattering factors are computed from Slater-type wave functions tabulated by Thakkar (Koga *et al.*, 1999 ▸). The methyl H atoms were constrained to tetra­hedral geometry, with bond lengths of 1.099 Å and displacement parameters set to 1.5 times the average *U* value of the methyl C atom, but no torsion angles were refined. The hy­dride H atom was refined anisotropically in order to distinguish it from other H atoms and to refine its occupancy factor; the mean-square atomic displacements found were: 0.07, 0.10, and 0.15. No positional parameter dif­ferences of more than 2σ were seen between the *SHELX* and *MoPro* refinements.

## Results and discussion

### Description of the X-ray structure

**Figure 1:**
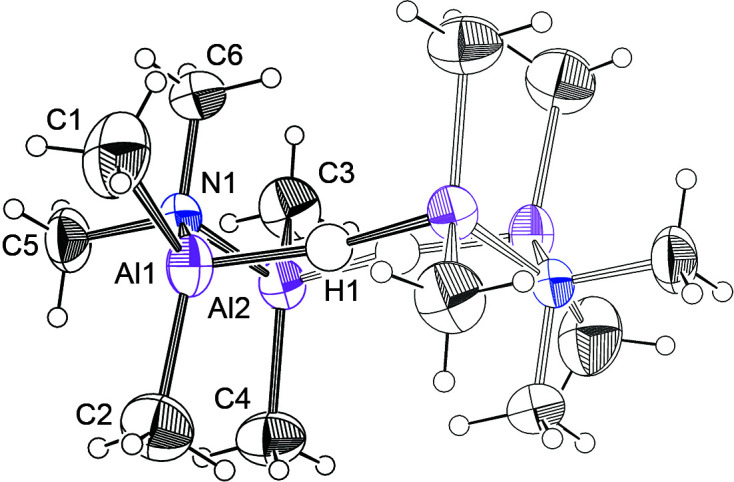
Displacement ellipsoid plot of the title com­pound, with ellipsoids at the 50% probability level, except that the methyl H atoms are plotted at an arbitrary scale. The asymmetric unit is highlighted in bold.

Fig. 1 ▸ shows atomic displacement ellipsoids for the asymmetric unit and the atom numbering. The title com­pound crystallizes as eight-membered rings with hy­dride H atoms joining two Me_2_Al–Me_2_N–Me_2_Al moieties. Overall, the mol­ecule has a chair conformation. The Al and hy­dride H atoms are essentially coplanar, with the H atoms just 0.06 (2) Å from the central plane of the four Al atoms, and the N atoms displaced 0.841 (2) Å above and below this Al_4_ plane. The dihedral angle between the Al_4_ central plane and the Al—N—Al edge plane is 54.3 (1)°, a little larger than the comparable angle of 49.2° in cyclo­hexane. Methyl groups C1, C3 and C5 are equatorial, and methyl groups C2, C4, and C6 are axial. The Al—N distances are normal, at 1.941 (3) and 1.941 (4) Å, while the Al—H distances are 1.657 (19) and 1.692 (19) Å. The inter­nal angles at the Al atoms are 95.8 (7) and 96.8 (7)°, while at the N atom, the inter­nal angle is 115.49 (10)°. The Al—H—Al angle deviates significantly from linearity, at 153 (1)°, with the H atoms moved towards the center of the ring, reducing the H⋯H distance to 2.52 (4) Å, close to the sum of the van der Waals radii for the H atoms.

### Supra­molecular features and Hirshfeld surface analysis

**Figure 2:**
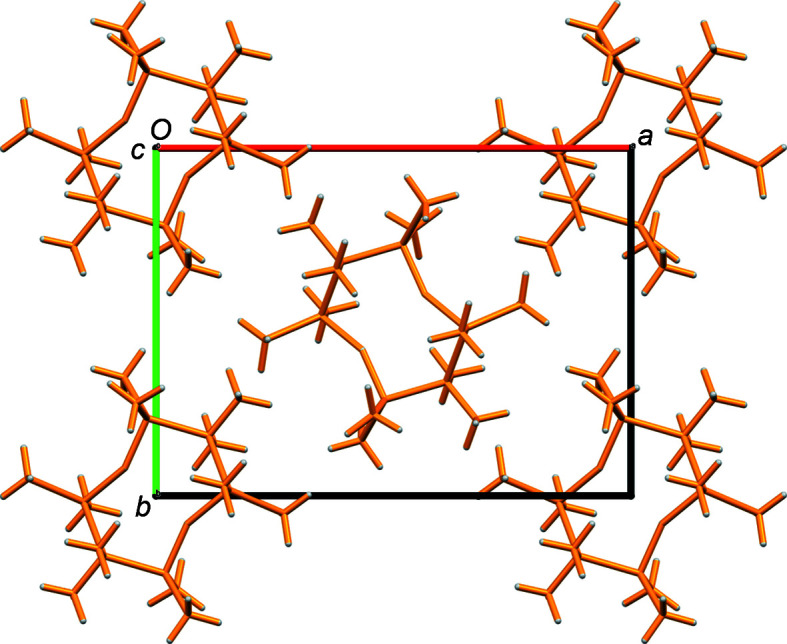
Packing diagram showing the projection down the *c* axis.

**Figure 3:**
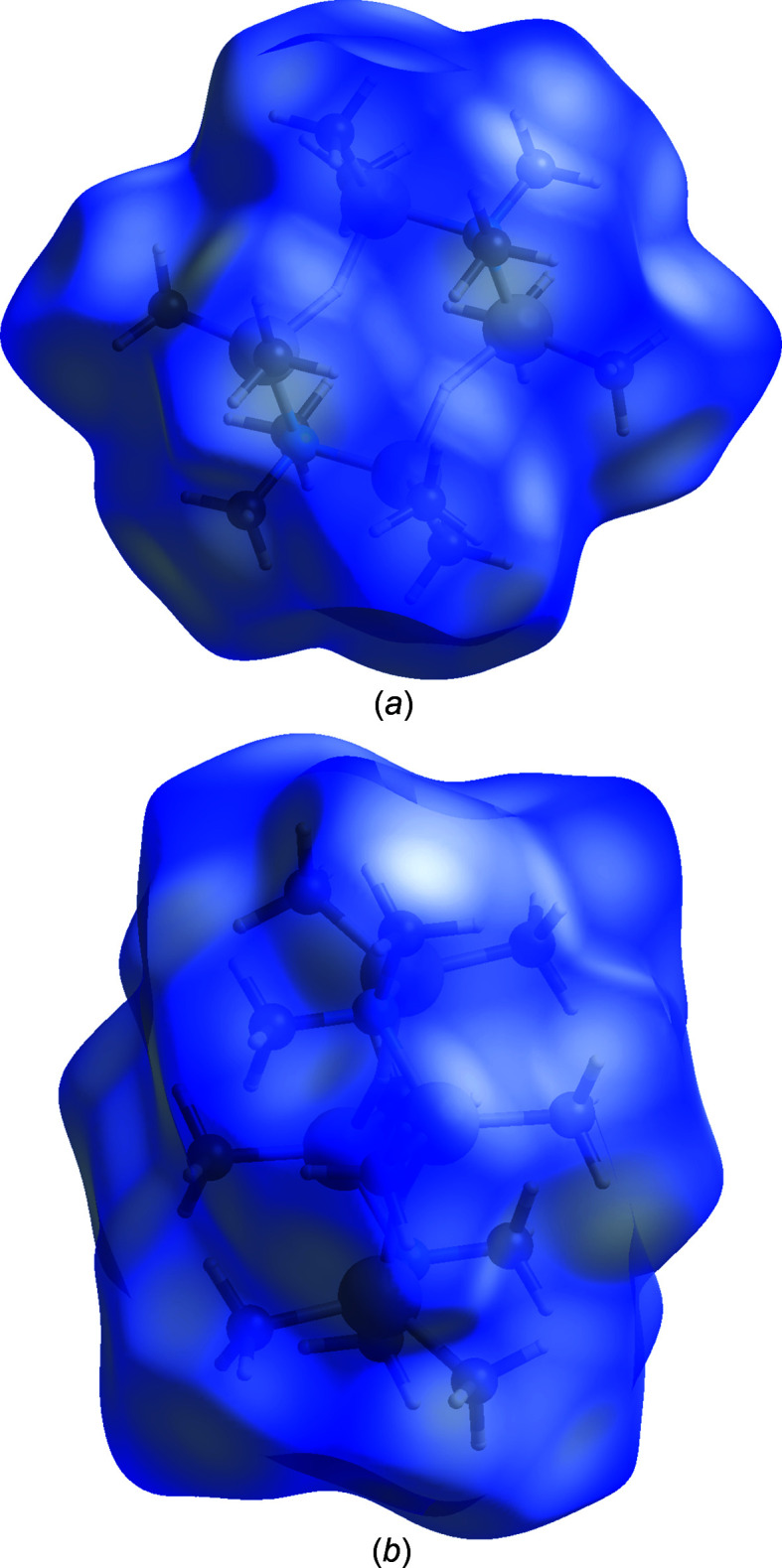
Hirshfeld *d*_norm_ surface (*a*) near the normal to the Al plane and (*b*) perpendicular to the view in part (*a*).

The mol­ecules pack in a centered arrangement with regard to the unit cell, as shown in Fig. 2 ▸. All the shortest inter­molecular contacts are due to H⋯H contacts between methyl groups, with H⋯H contact distances greater than twice the van der Waals radius for hydrogen. The mol­ecule was analyzed by the Hirshfeld procedure (Spackman *et al.*, 2009 ▸; Tan *et al.*, 2019 ▸) using *CrystalExplorer* (Turner *et al.*, 2017 ▸). The *d*_norm_ plots in Figs. 3 ▸(*a*) and 3(*a*) are all blue, again indicating no contacts less than the sum of the van der Waals radii. The surface tends to be flattened at the methyl groups. The fingerprint plot is featureless, in line with the lack of strong inter­molecular inter­actions, and all contacts at the surface are H⋯H contacts. Inter­molecular inter­action energies calculated with *CrystalExplorer* are given in Table 2 ▸. As can be seen, the dominant inter­actions are due to dispersion forces between the H atoms, with only minor contributions from Coulombic, polarization, and exchange–repulsion forces.

### Database survey

**Figure 4:**
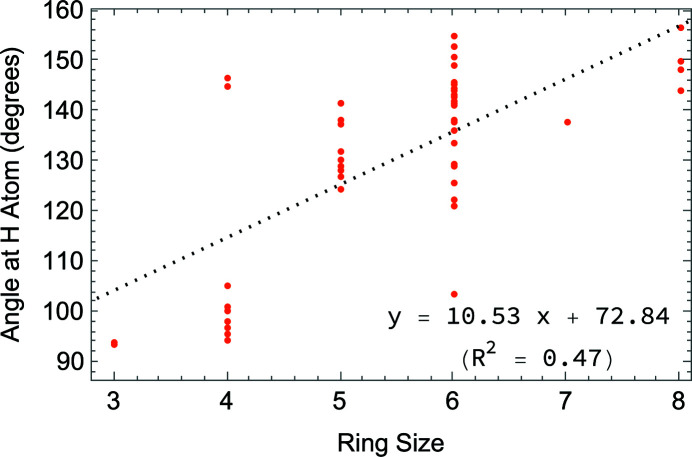
Chart showing the variation of hy­dride bond angles with ring size.

There are over 7000 crystal structures with a metal hy­dride bridge in the Cambridge Structural Database (CSD; Groom *et al.*, 2016 ▸). Of these, 52 hits were found that contain a singly-hy­dride-bridged pair of Al atoms and for which atomic coordinates were available. Six of these cases contained μ_3_- or μ_4_-bridging hy­drides, while the remaining 46 hits, with 64 hy­dride geometries, contain a single μ_2_-bridging hy­dride atom. The Al—H distances range from 1.46 to 1.90 Å, with a mean of 1.73 Å. The Al—H distances in the present structure of 1.657 (19) and 1.692 (19) Å fall at the center of this range. The angles at the hy­dride anion vary widely, from 94 to 180°. The hy­dride bond angle is clearly more flexible than the typical bond angle between normal two-center covalent bonds. One factor affecting this angle must be that the hy­dride bridge is often part of a ring of atoms that contain the Al—H—Al moiety, with ring sizes varying widely. The chart in Fig. 4 ▸ based on 56 cyclic structures shows a rough correlation between the hy­dride angle and the ring size, with the smallest angles generally occurring when the Al—H—Al bridge is in a three- or four-membered ring, and the larger angles being associated with larger ring sizes. (Seven of the other structures were acyclic and one had a very large ring which was not plotted.) A further factor in the wide range of bond angles observed is the presence of many Al cluster com­pounds, for which the bonding pattern is complicated. Hydride bond angles for eight-membered rings, for which there should be few stereochemical constraints, as in the present structure, range from 144 to 157°. The Al—H—Al bond angle in the present structure of 153 (1)° falls nicely in this range.

### Theoretical structure calculations

Density functional theory (DFT) calculations were per­formed on the neutral gas-phase C_12_H_38_Al_4_N_2_ building unit, at the unrestricted B97-2/aug-cc-pvDZ level using *GAUSSIAN16* (Frisch *et al.*, 2019 ▸). The optimized structure and charges are archived at the NOMAD repository (doi: 10.17172/NOMAD/2022.08.27-1). Previous work found that this level of theory gave the best description of the geometry and thermochemistry of Al_*n*_N_*m*_ and Al_*n*_H_*m*_ clusters (Lou­khovitski *et al.*, 2016 ▸, 2018 ▸). The gas-phase structure of the DFT calculations was expected to match the X-ray struc­ture, since the crystal inter­molecular forces are weak. As a check, DFT calculations were also made for dimers along the *z* axis and the *n*-glide plane directions, with insignificant changes in geometry and charge.

**Figure 5:**
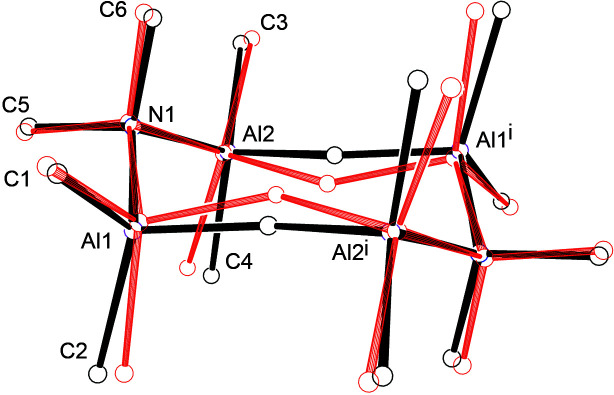
The X-ray (black) and DFT (red) structures displayed on common Al_4_-based axes. Methyl H atoms are not included. [Symmetry code: (i) −*x* + 1, −*y* + 1, −*z* + 1.]

**Figure 6:**
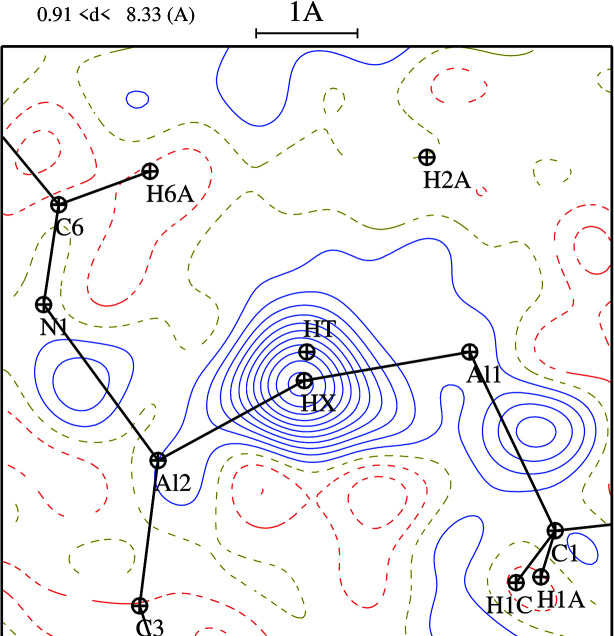
X-ray electron density in the Al—H—Al plane. The *F*_c_ values were from the refined structure less the hy­dride-atom contribution. HX gives the position of the hy­dride atom from the X-ray analysis and HT gives the DFT position. The contours are at 0.05 e Å^−3^.

We compare the X-ray structure and the fully optimized DFT structure in Fig. 5 ▸, where methyl H atoms have been excluded. The overall match is good, with bond angles between non-H atoms all agreeing within 1–2°, and bond lengths differing by no more than 0.03 Å, except for the Al—N bond length, which is 2.00 Å in the DFT structure *versus* 1.94 Å in the X-ray structure. Other differences include: (*a*) X-ray C—H distances show the expected shortening com­pared with those in the DFT structure (0.96 *versus* 1.10 Å), due to scattering of X-rays from the electron density, which for bonded H atoms is pulled into the bond and away from the nucleus; (*b*) torsion angles for the methyl groups are closer to the staggered conformation in the optimized structure, whereas the C2 and C3 methyl groups on the Al atoms are twisted 15 and 23° from the staggered conformation, presumably due to inter­actions in the crystal; (*c*) the DFT and X-ray hy­dride H-atom positions are 0.41 Å apart. The DFT Al—H—Al angle is 145°, smaller than the angle of 153 (1)° in the X-ray structure, while the hy­dride H atom is 0.27 Å above the Al_4_ plane in the DFT structure, compared with 0.06 (2) Å in the X-ray structure. The differences are illustrated in Fig. 6 ▸, which also shows an *F*_o_–*F*_c_ Fourier synthesis where the hy­dride contribution to *F*_c_ has been subtracted out. We can understand the hy­dride bridge in simple terms as a bent 3*c*—2*e* bond where both electrons come from the H^−^ ion, as reviewed recently by Parkin (2019 ▸). Presumably, much of the difference between the hy­dride geometry from the DFT calculations and from the X-ray crystal structures is again an artifact due to the electron density from the bridging H atom being pulled into the two bonds.

### Charge density analysis: X-ray and theoretical

#### X-ray charge analysis

The structure was refined by the conventional independent atom model (IAM) using *SHELXL2018* (Sheldrick, 2015 ▸). These are the parameters given in the CIF file associated with this article. The resulting IAM model was then refined along with valence shell population parameters by *MoPro* (Jelsch *et al.*, 2005 ▸). Scattering factors used were of the form *f* = *f*_core_ + *p*κ^3^ *f*_val_, where *p* is a refined parameter, constrained to be the same for chemically equivalent atoms and by a neutrality requirement, and κ is the radial expansion/contraction parameter, set at 1.16 for all methyl H atoms and at 1.00 for the other atoms, as suggested by *MoPro*. Refinement of the positional and displacement parameters and of the *p* values was carried out in ten alternating cycles. *MoPro R* values fell from *R*_1_(all) = 0.0544 to 0.0489, and *R*_w_ from 0.1270 to 0.1082. For just nine extra variables, this is a statistically significant drop, according to Hamilton’s *R*-factor significance test (Hamilton, 1965 ▸). The *R* values for *MoPro* were somewhat different from those in *SHELXL*, mainly due to the use of different scattering factors and a slightly different model. Partial charges derived from the *p* values are shown in Table 3 ▸. A CIF file from the *MoPro* refinements is given in the supporting information.

We had fixed κ values at 1.16 for H and 1.00 for the other atoms, as we would not expect κ values to be well defined by refinements with our limited data set. The refined *p* values, however, are expected to be correlated with the κ values. We have therefore explored the effects of variations in κ for the methyl H atoms, κ(H), first by carrying out refinements to convergence with fixed κ(H) values ranging from 1.06 to 1.26, and then by refining κ values. Refinements indicated that κ(Al), κ(N) or κ(H^−^) did not differ significantly from unity, and so these values were fixed at unity. A sample refinement where only κ(H) and κ(C) were allowed to vary converged with κ(H) = 1.05 (1) and κ(C) = 0.98 (1), with slightly higher *R* values. Partial charges corresponding to the *p* values from this refinement are also given in Table 3 ▸. Variations between those obtained from the fixed and from the refined κ values can give an idea of the uncertainties in our partial charges.

In all refinements, the C—H bond lengths were reset to the neutron diffraction value of 1.099 Å for C*sp* ^3^ (Allen & Bruno, 2010 ▸), as is usual for such studies (Stewart, 1970 ▸; Meenashi *et al.*, 2020 ▸). Significant differences in occupancies were observed if C—H distances were left at the X-ray values of 0.96 Å, as used in the X-ray analysis. However, when a C—H distance of 1.078 Å was used, the values used in *CrystalExplorer*, no change was more than 1σ. The X-ray charges are not sensitive to small changes in the neutron C—H bond lengths.

#### Theoretical charge analysis

Although the total electron density is a well-defined qu­antity in quantum mechanics and density functional theory, there is no unique decomposition of the electron density into atom-centered domains, and there are many different atomic charge assignment methods, which can give qu­anti­tatively and even qualitatively different results (Contreras *et al.*, 2017 ▸). Therefore, one expects only qualitative agreement amongst the different calculation methods and with the experimental results. Results in Table 3 ▸ show atomic partial charge decompositions using Hirshfeld, CM5, Merz–Kollman electrostatic potential, and natural bond orbital (NBO) methods using the optimized structure. Only small (±0.01 e) differences in the results were found when calculations were conducted with atoms fixed to the crystallographic coordinates, or in the presence of dimers along the *z* axis and glide-axis directions.

The variations between these predictions can be explained as follows:

(i) The Hirshfeld method (also known as a stakeholder or shareholder method) assigns the total electron density to atoms proportional to the relative neutral pro-atomic density; Hirshfeld charges are conceptually most similar to those from crystallographic analysis, but tend to underestimate the magnitude of the charge.

(ii) Charge Model 5 (CM5) adds an empirical correction to the Hirshfeld charges to reproduce experimental dipole moments. Although the CM5 training set included Al-con­taining com­pounds, the parameterization did not consider Al—N or Al—H—Al bonds, and so the empirical correction parameters may not be applicable for all of the types of atoms in our structure. Indeed, we see the largest discrepancies with the charge on the hy­dride.

(iii) Electrostatic potential (ESP) methods assign point charges to the atoms that best reproduce the mol­ecular electrostatic potential; we used the Merz–Kollman (MK) algorithm, although other variations exist (Francl & Chirlian, 2000 ▸). A general problem with ESP methods is that atoms buried within the inter­ior of a mol­ecule may be assigned non-physical partial charges as they have minimal contribution to the electrostatic potential surface. This could be an issue in our present application, as both the van der Waals spheres of the hy­dride H atoms and the Al atoms (surrounded by methyl groups) are obscured.

(iv) Natural Bond Orbital (NBO) methods express the wavefunction in terms of maximally localized atomic orbital-like basis functions whose core orbitals are close to doubly occupied and whose valence orbitals have single occupancy. This is conceptually most similar to the way formal charges are assigned when analyzing Lewis structures (McArdle, 2019 ▸). As such, they may overemphasize charge transfer.

#### Results

Despite the variations between the various theoretical methods, the data shown in Table 3 ▸ indicate qualitative agreement of the partial charges obtained from the *MoPro* refinements and by the DFT calculations. Both the experimental and all theoretical assignments suggest sub­stantial positive charges on the Al atoms, negative charges of about half an electron on the N and bridging H atoms, and negative charges on the C(Al) atoms that are much more negative than on the C(N) atoms. There are small positive charges on the methyl H atoms. These results are consistent with the Al—N and Al—C electronegativity differences of 1.0 and 1.5, which would indicate a polar Al—N and a more polar Al—C bond. The negative charge found for the bridging H atom is consistent with its characterization as a hy­dride. The *MoPro* refinement distinguishes between charges on the methyl H atoms on the Al and N atoms, whereas the charges estimated from the DFT calculations do not distinguish between these H atoms, although the total charges assigned to the Al-bound and N-bound methyl groups are different in each of the DFT calculations. A distinction between the Al-bound and the N-bound methyl groups might be expected on chemical grounds. The limited Cu *K*α resolution of the data used in this study forces the *MoPro* results to be limited to the spherical independent atom model, and the charges are not as well defined as we would wish; use of data collected with a shorter wavelength would have allowed a more sophisticated model by the *MoPro* program.

## Supplementary Material

Crystal structure: contains datablock(s) I, global. DOI: 10.1107/S2053229622011391/wp3031sup1.cif

Structure factors: contains datablock(s) I. DOI: 10.1107/S2053229622011391/wp3031Isup2.hkl

Mopro cif Table. DOI: 10.1107/S2053229622011391/wp3031sup3.txt

CCDC reference: 2222230

## Figures and Tables

**Table 1:** Experimental details

Crystal data	
Chemical formula	[Al_4_(CH_3_)_8_(C_2_H_7_N)_2_H_2_]
*M*_r_	318.36
Crystal system, space group	Monoclinic, *P*2_1_/*n*
Temperature (K)	295
*a*, *b*, *c* (Å)	14.175 (13), 10.378 (11), 7.692 (7)
β (°)	90.74 (4)
*V* (Å^3^)	1131.5 (19)
*Z*	2
Radiation type	Cu *K*α
μ (mm^−1^)	1.83
Crystal size (mm)	0.48 × 0.40 × 0.17

Data collection	
Diffractometer	Picker 4-circle diffractometer
Absorption correction	Gaussian (Busing & Levy, 1957 ▸); 8 × 8 × 8 grid
*T*_min_, *T*_max_	0.46, 0.71
No. of measured, independent and observed [*I* > 2σ(*I*)] reflections	3367, 1582, 1352
*R*_int_	0.068
θ_max_ (°)	58.0
(sin θ/λ)_max_ (Å^−1^)	0.550

Refinement	
*R*[*F* ^2^ > 2σ(*F* ^2^)], *wR*(*F* ^2^), *S*	0.039, 0.110, 1.07
No. of reflections	1582
No. of parameters	93
H-atom treatment	H atoms treated by a mixture of independent and constrained refinement
Δρ_max_, Δρ_min_ (e Å^−3^)	0.18, −0.20

Computer programs: Corfield & Gainsford (1972 ▸), Corfield *et al.* (1973 ▸), *SHELXL2018* (Sheldrick, 2015 ▸), *ORTEPIII* (Burnett & Johnson, 1996 ▸), *ORTEP-3* (Farrugia, 2012 ▸), and *publCIF* (Westrip, 2010 ▸).

**Table 2:** Inter­molecular inter­action energies in kJ mol^−1^ calculated with *CrystalExplorer* using electron density calculated with B3LYP/6-31G(d,p) Electronic (*E*_ele_), polarization (*E*_pol_), dispersion (*E*_dis_), and repulsion energies (*E*_rep_) are scaled with factors 1.057, 0.740, 0.871, and 0.618, respectively, when combined to form the total energy of inter­action. *R* is the distance between mol­ecular centroids, in Å.

	*N*	Sym	*R*	*E*_ele_	*E*_pol_	*E*_dis_	*E*_rep_	*E*_total_
1	2	*c trans*	7.69	−4	−2	−28	8	−26
2	4	*n*-glide	9.55	0	−1	0	0	−1
3	4	*n*-glide	9.63	−2	−1	−21	7	−18
4	2	**b* *trans**	10.38	−4	−1	−14	2	−16

**Table 3:** Partial charges for the chemically independent atoms in Al_4_(CH_3_)_6_N_2_H_2_ The *K* values for the two *MoPro* results are given in the text.

Method	*MoPro*		Theoretical			
Atom	Fixed *K*	Varied *K*	Hirshfeld	cm^5^	ESP	NBO
Al	1.01 (9)	1.48 (10)	0.45	0.29	0.91	1.72
N	−0.42 (4)	−0.36 (4)	−0.14	−0.42	−0.05	−1.09
H(bridging)	−0.45 (5)	−0.59 (5)	−0.13	0.00	−0.34	−0.52
C(Al meth­yl)	−0.95 (4)	−1.01 (5)	−0.27	−0.44	−0.81	−1.22
C(N meth­yl)	−0.48 (5)	−0.50 (5)	−0.04	−0.19	−0.41	−0.34
H(Al meth­yl)	0.27 (2)	0.24 (2)	0.02	0.10	0.15	0.21
H(N meth­yl)	0.07 (2)	0.02 (3)	0.05	0.13	0.14	0.19
